# Atypical Appendicitis in the Elderly

**DOI:** 10.7759/cureus.22495

**Published:** 2022-02-22

**Authors:** Polina Gaisinskaya, Taylor VanHelmond, Oscar L Hernandez

**Affiliations:** 1 Internal Medicine, Florida Atlantic University, Boca Raton, USA; 2 Internal Medicine, Florida Atlantic University Charles E. Schmidt College of Medicine, Boca Raton, USA

**Keywords:** hernia in elderly, elderly individuals, amyands hernia, hernia, appendicitis

## Abstract

Amyand’s hernia is an extremely rare condition in which the appendix is positioned in the inguinal hernia sac. Acute appendicitis is much less common in this situation, especially in the elderly population, and few reports are found in the literature. Whether the presence of the appendix within the inguinal sac increases the chance of developing appendicitis or the relationship is coincidental is unclear. Majority of cases reported are in the male pediatric population. The varying surgical approaches are entirely case-specific without much standardization. Perioperative planning remains difficult in all cases as this condition is exceedingly rare, especially outside of the pediatric population. We present such a case with the hope that we increase awareness about this uncommon condition, in which preoperative diagnosis and planning remains difficult.

## Introduction

Inguinal hernias account for 75% of abdominal wall hernias. Incarceration of the appendix in the inguinal sac is known as Amyand’s hernia and can present with or without acute appendicitis. It was first described by Claudius Aymand in 1735 in an 11-year-old boy, and this condition remains rare with an incidence of around 0.5-1% [1]. Even rarer is the presentation of acute appendicitis within the inguinal sac, with an incidence of around 0.1% [1]. Differential diagnosis may include strangulated hernia, strangulated omentocele, Richter’s hernia, testicular tumor with hemorrhage, acute hydrocele, inguinal adenitis, and epididymitis [2]. Whether the appendix found within the inguinal sac is an incidental finding or there is a casual relationship between the cases remains unclear. It is possible for these patients to develop abscess formation as well presenting as inguinal masses.

We presented this rare case at the American College of Gastroenterology conference as a poster in October 2021.

## Case presentation

**Figure 1:**
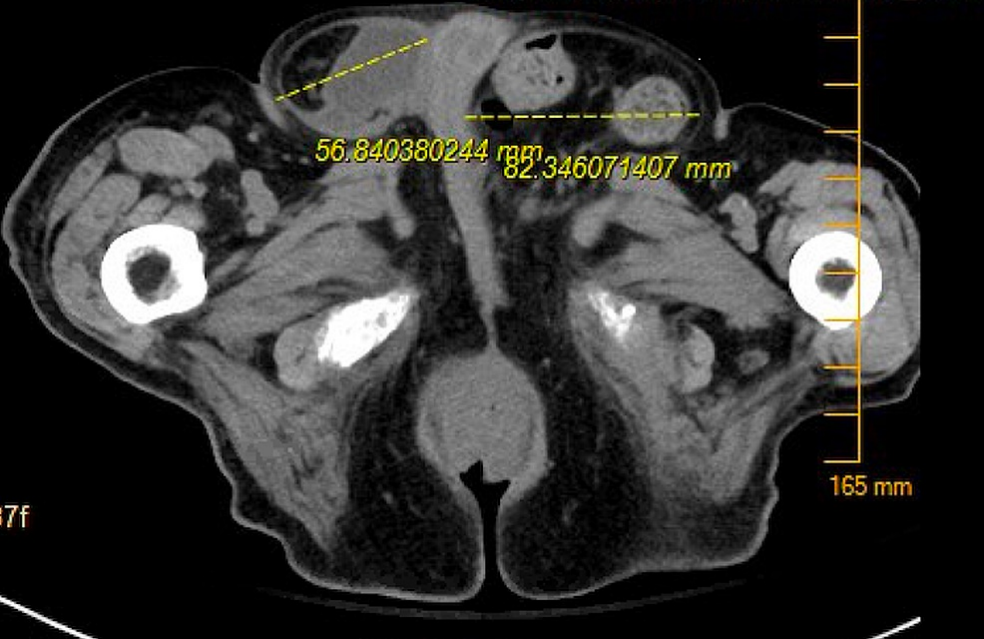
Computed tomography (CT) scan done at the time demonstrated a large left-sided hernia containing the sigmoid colon without evidence of stranding, inflammation, or obstruction. The right side was also noted to have a large hernia with the cecum and appendix in the sac associated with stranding and some pericolonic fluid collection.

A 97-year-old male with a history of advanced dementia, benign prostatic hypertrophy, urinary stones, and 10-year history of bilateral non-reducible inguinal hernias extending down into his scrotum who presented to the emergency department (ED) with right-sided abdominal pain. In the ED, the patient was noted to be hemodynamically stable. Physical examination was notable for soft, non-distended abdomen with significant tenderness to palpation along the right lower quadrant, and two large bilateral inguinal hernias, and the patient was non-cooperative to reduction at the time. Lab work was without any notable leukocytosis or lactic acidosis. Computed tomography (CT) scan performed at the time demonstrated a large left-sided hernia containing the sigmoid colon without evidence of stranding, inflammation, or obstruction. The right side was also noted to have a large hernia with the cecum and appendix in the sac associated with stranding and some pericolonic fluid collection (Figure 1). General surgery was consulted at this time with concerns for incarcerated right-sided inguinal hernia. The patient was empirically started on antibiotic treatment. Informed consent was obtained from his power of attorney, and he underwent open right inguinal hernia repair with mesh placement and appendectomy with intraoperative findings of reducible right direct and indirect inguinal hernia and inflammatory changes of the appendix indicative of likely appendicitis. The left was also repaired prophylactically with mesh. The patient did well post-operatively and was discharged back to his skilled nursing facility with instructions to follow up with surgery for pain control and no further testing.

## Discussion

Amyand’s hernia is present three times more likely in the pediatric patient due to a patent processus vaginalis and appears more commonly in males [2]. It typically presents on the right side based on the anatomy, but it has also been presented on the left due to gut malrotation, situs inversus totalis, and a very mobile cecum [3]. Because patients may present with nonspecific symptoms, this remains a difficult diagnosis to make preoperatively and hence is mainly found intraoperatively during surgical exploration of a complicated inguinal hernia [4]. A classification system designed to diagnose and treat Amyand’s hernia was created and is called the Losanoff and Basson’s criteria. It consists of four different types: type 1, a normal appendix in the hernia sac; type 2, a hernia with acute appendicitis; type 3, a hernia with acute appendicitis and abdominal sepsis; and type 4, acute appendicitis with related or unrelated abdominal pathology [5].

With the increased use of CT scans, this diagnosis is more frequently being made preoperatively, allowing for both safer preoperative planning and intraoperative decision-making [2]. CT scans will typically demonstrate the appendix within the inguinal canal with or without inflammation (increased luminal diameter, fat stranding, fluid collection, associated cecal thickening, and presence of appendicolith in a few cases) [3]. With ultrasound playing a large role in diagnosis as well, you may see non-compressible, dilated, blind-ending bowel loops with a luminal diameter of more than 7.2 cm within the inguinal canal with or without inflammation [3]. Ultrasound may have additional benefits of being real-time and visualize certain features unavailable with the CT scan [4]. Recent cases have demonstrated that laparoscopy is becoming the leading choice for treatment based on staging of the hernia and inflammation of the appendix due to improvements in preoperative diagnosis with CT and ultrasound [5]. However, the use of mesh in repair of Amyand's hernia with appendicitis has been demonstrated to have bacterial seeding, mesh sepsis, recurrent hernia, and recurrent surgical site infections [6,7]. There are typically few complications associated with Amyand's hernia, though some serious complications such as necrotizing fasciitis have been documented [7]. In the treatment of Amyand's hernia, there is an increasing number of documented cases exploring the use of laparoscopic repair versus open, but more research is needed in the future to determine the specific indications of open versus laparoscopic repair with consideration of staging the appendectomy first with later hernia repair [8]. In a large retrospective analysis, the inflammatory status of the appendix in these cases was used to determine the surgical approach and hernia repair required for each case [9]. We hope that presentation of our case helps guide future diagnostic studies and treatment options in future patients.

## Conclusions

Amyand's hernia, although quite rare, should remain on the differential in patients presenting similarly to ours. While the patient will ultimately require surgical interventions, it is important to keep in mind the role of pre-operative imaging that may play a big role in management of such patients prior to their surgery and lead to improved preoperative planning and intraoperative decision-making. Given its rarity, it is difficult to standardize an approach applicable to most cases. Especially in the geriatric population, perioperative planning will aid in choosing the least invasive approach, which can optimize post-operative pain control and prevent delirium in this specific patient population. The use of perioperative imaging may be the key to personalizing each case to the best of the physician’s abilities. Early diagnosis and awareness of such condition will also aid in timely intervention with proper planning before complications may arise. We hope that by presenting this case we may allow everyone, including the emergency medicine physician and hospitalists, to keep this condition in mind with prompt surgical referral.
